# Nurse Staffing and Emergency Department Crowding: A 10-State Study

**DOI:** 10.1097/MLR.0000000000002379

**Published:** 2026-09-25

**Authors:** K. Jane Muir, Linda H. Aiken, Karen B. Lasater, Matthew D. McHugh, Daniela Golinelli

**Affiliations:** * Center for Health Outcomes and Policy Research, School of Nursing, University of Pennsylvania, Philadelphia, PA; † Department of Emergency Medicine, Perelman School of Medicine, University of Pennsylvania, Philadelphia, PA; ‡ The Leonard Davis Institute of Health Economics, University of Pennsylvania, Philadelphia, PA

**Keywords:** emergency department, staffing, length of stay, crowding

## Abstract

**Background:**

The purpose of this study was to determine the relationship between nurse staffing inadequacy in the emergency department (ED) and inpatient units on objective ED crowding measures.

**Methods:**

Three datasets from 2024 were linked: the Penn Nurses4All survey, CMS Hospital Compare, and the AHA Annual Hospital Survey. Nurses4All provided measures of nurse staffing inadequacy. The proportion of nurses reporting staffing inadequacy was the main exposure variable. Crowding measures included the percentage of patients who left the ED without being seen and patient length of stay (in minutes) for patients with and without psychiatric/mental health presentations. The analysis included hospital-level regression models adjusted for hospital characteristics.

**Results:**

The sample included 410 hospitals in 10 states. A 1 SD increase in ED nurses reporting staffing inadequacy was associated with higher hospital left without being seen rates (β=0.4, 95% CI: 0.2–0.6, *P*<0.001), longer lengths of stay for ED patients (β=8.5, 95% CI: 3.5–13.6, *P*<0.001), and patients with psychiatric/mental health concerns in the ED (β=18.4, 95% CI: 3.6–33.1, *P*<0.05). A 1 SD increase in inpatient nurses reporting staffing inadequacy was associated with longer ED lengths of stay for patients with psychiatric/mental health concerns (β=18.0, 95% CI: 5.8–30.3, *P*<0.01).

**Conclusion:**

Staffing a sufficient number of nurses in both the ED and inpatient units is a promising strategy to alleviate ED crowding.

In 2007, the National Academy of Medicine published “Hospital-Based Emergency Care: At the Breaking Point,” declaring emergency department (ED) crowding a public health emergency given threats to timely and high-quality patient care.^1^ Almost 20 years later, evidence demonstrates that hospital ED boarding has increased in frequency and duration and is associated with poor patient outcomes, including prolonged hospital stays^2^ and adverse events such as medical errors.^3,4^ Crowding contributes to delays in patients getting admitted and discharged, and patients may be boarded in the ED for hours to days as they wait for an inpatient bed to become available.^5,6^

Several interventions to reduce ED crowding have been tested in single-site studies, including implementing provider-in-triage models^7^ in waiting rooms, using rapid medical evaluation spaces,^8^ and expanding the number of ED beds in hospitals.^9,10^ These interventions have demonstrated mixed success in reducing crowding.^7,8^ The Agency for Healthcare Research and Quality Summit to Address Emergency Department Boarding^10^ concludes that provider-in-triage models, building larger EDs, ambulance diversion, and expanding telehealth options have not been effective in reducing hospital crowding.

Janke and colleagues demonstrate that boarding for >24 hours has increased since before the pandemic, with 40% of admitted patients boarding in the ED for longer than the maximum of 4 hours recommended by the Joint Commission.^11–13^ A cohort study by Roussel and colleagues^14^ identified that patients who board overnight are more likely to die in the hospital compared with patients admitted to a specialty ward (15.7% vs. 11.1%). Finally, it costs about $1000 more per patient for an admitted patient to board in the ED than in a dedicated medical-surgical unit.^15^

Two significant barriers identified in the literature include a prevailing focus on the ED as the primary target for the crowding intervention and an under-recognition that patient throughput relies on having enough nurses in the hospital to facilitate patient flow.^10,16^ Minimal research has evaluated the association of nurse staffing adequacy with ED crowding outcomes.

## CONCEPTUAL FRAMEWORK

Theoretically, the Asplin input-throughput-output model^17^ (Supplemental Fig. 1, Supplemental Digital Content 1, https://links.lww.com/MLR/D241) demonstrates how ED crowding is driven by inefficiencies and delays not just in hospital EDs but also in the inpatient setting. Nurses in the ED and inpatient settings facilitate patient throughput at all stages of the Asplin model, including the input (ED triage and care), throughput (care delivery during ED and inpatient stay), and output (patient education, discharge, and follow-up). To date, it is unknown whether and to what extent nurse workload, specifically whether nurses are adequately staffed in their workplace, is associated with the throughput of patients in Asplin’s theoretical model and therefore ED crowding.

### Literature Gap

ED crowding is a systemic issue, not just driven by inefficiencies in the ED, but also delays and resource inadequacies in the inpatient setting.^17^ A study using 2016 nurse staffing data identified that hospitals with safer medical-surgical staffing ratios (<5:1 vs. ≥5:1) have shorter ED patient boarding times^18^. No studies have identified associations between nurse staffing adequacy in the ED, inpatient settings, and the overall hospital level.

Over 2 decades of evidence demonstrate that nurse workload^19,20^ is associated with patient outcomes, and nurse job outcomes, including burnout, yet it is unknown if there is a relationship with ED crowding, including left without being seen rates and ED length of stay. Nurses are the primary surveillance system in hospitals, and their reports on patient care quality and safety are associated with objective patient care quality outcomes.^21^

### Study Objectives

While many factors contributing to ED crowding are not easily modifiable, like the difficulties of placing many long-term care and psychiatric patients in other, more appropriate care settings, acute care hospital nursing resources are. Hospitals that invest in safer patient-to-nurse staffing ratios experience shorter inpatient lengths of stay and fewer hospital readmissions.^22,23^ The relationship between hospital nurse staffing and ED crowding is an understudied possible solution to the intractable problem of ED crowding. The primary objective of this study was to determine the association between hospital nurse staffing and ED crowding. We hypothesized that hospitals with more nurses reporting staffing inadequacy would be associated with worse ED crowding measures.

### Exploratory Objective

Nurses may also be primary informants to the severity of crowding in their hospital, allowing them to modify aspects of patient throughput by facilitating timely discharges, modifying patient care spaces, bringing in “on-call” nurses when necessary, and collaborating with physicians to advance care plans.^24^ An exploratory secondary objective of this study was to determine whether nurses' reports on boarding are associated with objective measures of crowding, given that they are informants of other hospital-related factors such as the quality of patient care.^21^ We also hypothesized that nurse-reported boarding would be associated with objective ED crowding measures.

## METHODS

### Study Design

This cross-sectional study used 3 datasets linked and merged through a common hospital identifier: the 2024 Penn Nurses4All Study of nurses licensed to work in 10 states, the 2024 Hospital Compare timely and effective care data, and the American Hospital Association Annual Hospital Survey.^25^ The Penn Nurses4All survey methodology is discussed in detail elsewhere.^26,27^ The University of Pennsylvania Institutional Review Board approved this study.

The Penn Nurses4All survey was a multistate electronic survey of registered nurses licensed to work in 9 states (CA, FL, IL, LA, NJ, NM, NY, OR, WA) and a mail survey of nurses in PA (because emails were not available).^26^ The survey was conducted between December 2023 and March 2024. All RNs were surveyed in the 9 states, and a random sample of RNs in PA was surveyed because of the high cost of mail surveys. The survey went to all nurses without regard to work setting or whether they were currently employed. Nurses reported on the conditions of nursing care and nursing practice in their employment settings and indicated in the survey the identity of their employer. Nurses in hospitals were aggregated to their employing hospitals, resulting in a study of 410 hospitals. The full details of the Nurses4All survey have been published elsewhere.^27,28^

Our approach surveys nurses directly rather than sampling hospitals. This method reduces hospital nonresponse bias that may result from adopting a cluster sampling approach, which requires participation of the sampled hospitals.^28^ As compared with most other surveys of nurses, we ask nurse respondents to provide the name of their employer to permit results to be examined at the hospital level, which is the point for potential intervention. The Nurses4All sample covers over 95% of the hospitals located in the 10 study states.

### Setting and Sample

The hospital sample for this study was derived from linking 3 distinct datasets. Of the hospitals that could be linked across the 3 data sources, by design, we included those that have at least 3 nurses responding to the Penn Nurses4All and at least 2 ED nurses responding.

### Measures

#### Independent Variable: Nurse Staffing

Nurse staffing was measured as the proportion of nurses within each hospital reporting inadequate staffing. This variable was generated from the survey question: present in my current job [rank from strongly agree (1) to strongly disagree (4)]: enough staff to get the work done. The measure of nurse staffing adequacy has been used for over 15 years as a valid measure of nurse workload in hospitals in the United States and internationally across multiple studies.^29–31^ This measure of nurse workload is superior to the FTE measure from the American Hospital Association that may include nurses who do not carry a direct care assignment (eg, nursing supervisors) or who may practice in outpatient settings. The measure demonstrated good reliability when assessed across nurses working within the same hospital, with an intraclass coefficient [ICC(2)]=0.60.

The measure was dichotomized by coding “inadequate staffing” as the response options “disagree” and “strongly disagree.” The proportion of nurses within a hospital reporting inadequate staffing served as a hospital-level measure of staffing inadequacy. We standardized the measure to have a mean of 0 and an SD of 1.

We developed 3 measures of nurse staffing: ED staffing inadequacy (n=1700 nurses), inpatient staffing inadequacy (inclusive of nurses in all hospital units except the ED; n=9857), and overall hospital nurse staffing inadequacy (inpatient and ED nurses; n=11,705). Models with only ED nurses had 410 hospitals (average 4 RNs per hospital, range: 2–19); inpatient-only models included 573 hospitals (average 17 RNs per hospital, range: 3–162); overall hospital models included 601 hospitals (average 19 RNs per hospital, range: 3–172). We also ran sensitivity analyses adjusting for missingness on the outcome variables using nonresponse weights (Supplemental File, Supplemental Digital Content 1, https://links.lww.com/MLR/D241).

#### Independent Variable: Frequent Emergency Department Boarding

The second independent variable explored was frequent ED boarding, reported by ED nurses. ED nurses in the Penn Nurses4All survey answered the question “how frequently does your ED board patients (ie, hold them in the ED due to a lack of available beds)?” on a Likert scale (never, daily, weekly, monthly). ED boarding was considered “frequent” if nurses reported daily boarding in their ED. Thus, this variable was dichotomous (frequent: yes or no) and measured as a proportion of ED nurses. This measure was also standardized for interpretation purposes. The distributions of both independent variables are provided in Figure 1.

**FIGURE 1:**
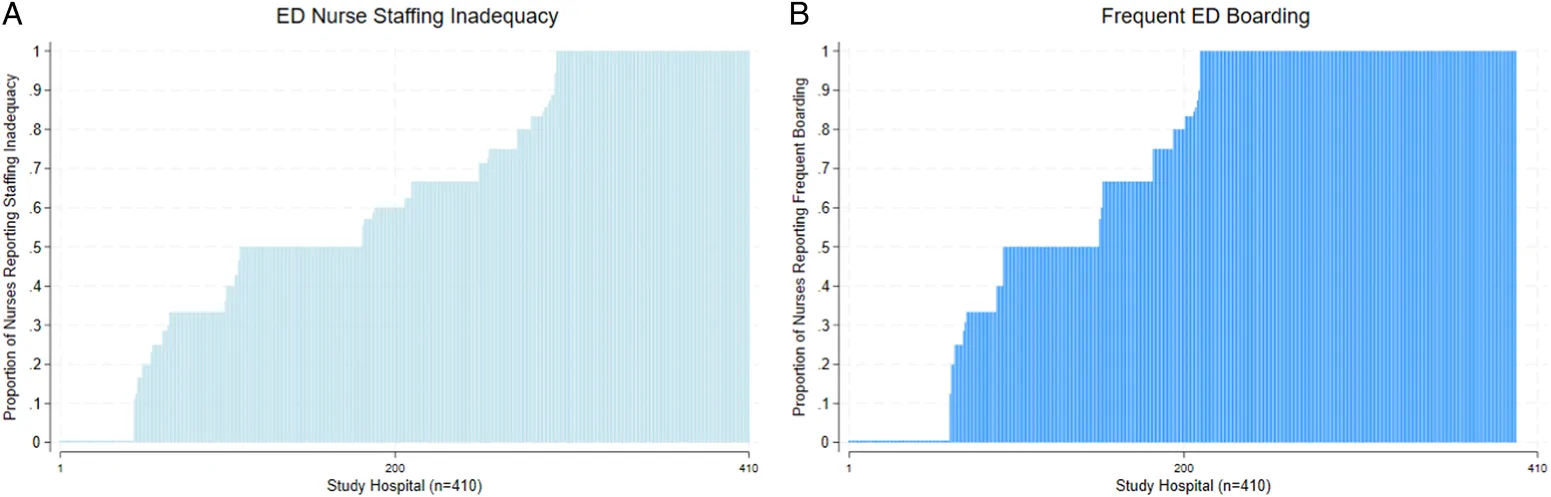
Variation in exposure variables Figure A describes the variation in the proportion of ED nurses reporting inadequate staffing. Figure B describes the variation in the proportion of ED nurses reporting frequent patient boarding.

#### Dependent Variables: Emergency Department Crowding

ED crowding was measured using the Hospital Compare timely and effective care files published in July 2024, representing data collection between October 2023 and September 2024.^5^ These hospital-level variables include the percentage of patients who left the ED without being seen (rate), the overall ED patient length of stay (minutes; excluding psychiatric/mental health patients), and the psychiatric/mental health patient ED length of stay (minutes). Although there is no national recommendation on hospital crowding metrics, the Joint Commission guidelines for hospitals outline a recommended left without being seen rate of <2%,^32^ and an ED length of stay of <4 hours. Other sources have recommended a goal of <6 hours for ED length of stay.^33^

#### Hospital Covariates

The hospital characteristics used for covariate risk adjustment were state, teaching status (nonteaching: no medical fellows/residents; minor teaching: <1 fellow/resident per 4 beds; major ≥1 fellow/resident to 4 beds), technology capabilities (indicating capacity to provide transplant services, yes or no), and annual ED patient volume, derived from the AHA Annual Hospital Survey.

### Data Analysis

#### Descriptive Statistics

We computed descriptive statistics, including means, percentages, and SDs for hospital characteristics, nurse staffing, and ED crowding measures.

#### Association of Independent Variables With Emergency Department Crowding

All study models were constructed at the hospital level, unadjusted and adjusted for hospital characteristics. First, we constructed linear regression models determining the association of hospital nurse staffing inadequacy with hospital left without being seen rates, ED length of stay for all patients (excluding those with psychiatric/mental health conditions), and ED length of stay for psychiatric/mental health patients. We produced 3 separate models assessing the association of ED, inpatient, and overall nurse staffing (each in 3 separate models) and ED crowding measures.

We then constructed hospital-level linear regression models determining the relationship between boarding and objective crowding measures, unadjusted and adjusted for hospital characteristics.

#### Projected Association of Emergency Department Crowding With Improving Nurse Staffing Adequacy

Using the β coefficients produced in our study models, we projected the additional minutes saved and additional patients seen if the proportion of nurse staffing inadequacy was reduced by 1 SD (ie, staffing was improved; Table 4). We repeated the same procedures using the hospital left without being seen rate to estimate the number of patients retained in care.

## RESULTS

The final sample included 410 hospitals in 10 states. Most hospitals had high technology capabilities (55%), and the average annual ED volume was 64,051 (SD=60,500; Table 1). On average, 58% of hospital nurses reported inadequate staffing (not enough staff to get work done) overall (average proportion=0.58; SD=0.22; IQR=0.45–0.73). In the inpatient setting, 58% of nurses reported inadequate staffing (0.58; SD=0.23; IQR=0.6–1.0), and in the ED, 61% of nurses reported inadequate staffing (0.61; SD=0.37; IQR=0.4–1.0).

**TABLE 1:** Hospital Characteristics (n=410 Hospitals)

Hospital characteristic	Outcome, n (%)
Proportion of nurses reporting inadequate staffing, mean±SD	
All nurses	0.58±0.22
Inpatient nurses	0.58±0.23
ED nurses	0.61±0.32
Hospital crowding measures, mean	
Left without being seen (%, SD)	2.8 (2.2)
ED length of stay (minutes, SD)	204.9 (51)
ED length of stay, psychiatric/mental health patients (minutes, SD)	323.8 (133)
Proportion of ED nurses reporting frequent boarding (minutes, SD)	0.68 (0.37)
Teaching status	
Not	150 (37)
Minor	155 (38)
Major	105 (25)
High technology capabilities	
Yes	224 (55)
No	186 (45)
Annual ED volume (no. patients seen), mean±SD	64,051±60,500
State, hospital	
CA	104 (25)
NY	60 (15)
PA	55 (13)
OR	44 (11)
FL	39 (10)
IL	36 (9)
NJ	27 (7)
WA	28 (7)
LA	9 (2)
NM	8 (2)

Percentages may not total 100 due to rounding.

ED indicates emergency department.

ED crowding measures (Table 1) include the left without being seen rate and the ED length of stay in minutes for patients with and without psychiatric/mental health conditions. On average, the hospital left without being seen rate was 2.8% (SD=2.2; IQR=1%–3%). The average ED patient length of stay was 205 minutes (3.4 h, SD=51 min, IQR=171–237 min), and the psychiatric/mental health patient length of stay was 324 minutes (5.4 h, SD=133 min, IQR=232–394 min). The proportion of ED nurses reporting frequent boarding was 0.68 (SD=0.37; IQR=0.5–1.0).

### Association of Nurse Staffing and ED Crowding

Hospital-level regression models determining the relationship between nurse staffing and ED crowding are provided in Table 2. Among ED nurses, the relationship between nurse staffing inadequacy and all crowding outcomes was statistically significant in both the unadjusted and adjusted models. In the adjusted models, a 1 SD increase in ED nurses reporting nurse staffing inadequacy was associated with a 0.4 percentage-point increase in the hospital left without being seen rate (β=0.4, 95% CI: 0.2–0.6, *P*<0.001), a 8.5-minute increase in ED patient length of stay (β=8.5, 95% CI: 3.5–13.6, *P*<0.001), and a 18.4-minute increase in psychiatric/mental health patient length of stay (β=18.4, 95% CI: 3.6–33.1, *P*<0.05).

**TABLE 2:** Linear Regression Models Describing the Association of Nurse Staffing Inadequacy and Measures of Hospital Crowding

	β coefficient (95% CI)		
Exposure Variable	Left without being seen (%)	ED length of stay*	ED length of stay^†^
ED staffing, unadjusted	0.3 (0.1–0.6)^‡^	11.7 (6.2–17.2)^§^	25.7 (11.2–40.2)^‡^
ED staffing, adjusted	0.4 (0.2–0.6)^‡^	8.5 (3.5–13.6)^‡^	18.4 (3.6–33.1)^‖^
Inpatient staffing, unadjusted	0.01 (−0.2 to 0.2)	4.0 (−0.6 to 8.6)	18.8 (6.7–30.9)^‡^
Inpatient staffing, adjusted	0.2 (−0.04 to 0.4)	2.5 (−1.8 to 6.8)	18.0 (5.8–30.3)^‡^
Overall staffing, unadjusted	0.1 (−0.1 to 0.3)	7.8 (3.4–12.3)^‡^	25.0 (13.5–36.4)^§^
Overall staffing, adjusted	0.3 (0.1–0.5)^‡^	5.8 (1.7–9.9)^‡^	22.7 (11.0–34.3)^§^

Length of stay is measured in minutes. Model covariates include state, technology capabilities, teaching status, and annual ED volume. The hospital sample across models includes ED staffing (n=410 hospitals), inpatient (n=573 hospitals), and all-nurse (n=601 hospitals).

^*^ Length of stay excluding psychiatric/mental health patients.

^†^ Length of stay for psychiatric/mental health patients.

^‡^ *P*<0.01.

^§^ *P*<0.001.

^‖^ *P*<0.05.

ED indicates emergency department.

Among inpatient nurses, a 1 SD increase in nurse staffing inadequacy was associated with an 18-minute increase in the psychiatric/mental health patient length of ED stay (β=18.0, 95% CI: 5.8–30.3, *P*<0.01). No other crowding measures were significantly associated with inpatient staffing in the unadjusted or adjusted models. In the models including all hospital nurses, a 1 SD increase nurse staffing inadequacy was associated with a 0.3 percentage-point increase in the hospital left without being seen rate (β=0.3, 95% CI: 0.1–0.5, *P*<0.01), a 5.8-minute increase in the overall ED patient length of stay (β=5.8, 95% CI: 1.7–9.9, *P*<0.01), and a 22.7-minute increase in psychiatric/mental health patient length of stay (β=22.7, 95% CI: 11.0–34.3, *P*<0.001).

### Exploratory Analysis: Boarding and Emergency Department Crowding

Hospital-level linear regression models determining the relationship between boarding and ED crowding are provided in Table 3. In the fully adjusted models, a 1 SD increase in the proportion of ED nurses reporting frequent ED boarding was associated with a 10-minute increase in overall ED length of stay (β=10.3, 95% CI: 5.2–15.3, *P*<0.01) and a 19-minute increase in the psychiatric/mental health patient length of stay (β=19.4, 95% CI: 5.0–34.0, *P*<0.01).

**TABLE 3:** Association of Nurse-Reported Boarding on Hospital Crowding

	β coefficient (95% CI)		
Exposure Variable	LWOBS	ED length of stay^*^	ED length of stay^†^
Nurse-report boarding			
Unadjusted	0.1 (−0.1 to 0.4)	15.8 (10.6–21.0)^‡^	20.0 (5.6–33.7)^§^
Adjusted	0.2 (−0.1 to 0.4)	10.3 (5.2–15.3)^‡^	19.4 (5.0–34.0)^§^

Model covariates include state, technology capabilities, teaching status, and annual ED volume. The hospital sample across models includes 397 hospitals with nurses reporting onboarding.

^*^ Length of stay excluding psychiatric/mental health patients.

^†^ Length of stay for psychiatric/mental health patients.

^‡^ *P*<0.001.

^§^ *P*<0.01.

ED indicates emergency department; LWOBS, left without being seen (rate).

### Projected Hospital Impact

The projected impact of the ED nurse staffing inadequacy on crowding outcomes for the average ED annual patient volume of 64,000 in our sample is provided in Table 4. Based on a 1 SD reduction in ED nurse staffing inadequacy, we estimate that hospitals seeing 64,000 patients a year will see an additional 2939 patients due to a reduction in overall ED length of stay. For psychiatric/mental health patient length of stay, the number of additional patients seen is 3765. For those left without being seen, a 1 SD reduction in ED nurse staffing inadequacy would retain ∼256 patients a year in hospitals for care. These are underestimates for the nation since they are derived from data from only 10 states, and the national results would likely be much higher.

**TABLE 4:** Estimated Additional Patients Seen With Reductions in ED Length of Stay and Left Without Being Seen Rates Due to Better Staffing

ED length of stay					
ED patient volume	Baseline ED LOS (min)	Improved LOS (min)	Additional care minutes	Additional patients seen
64,000	205	196	576,000	2939
Psychiatric/mental health patient ED length of stay					
ED patient volume	Baseline ED LOS	Improved LOS	Additional care minutes	Additional patients seen
64,000	324	306	1,152,000	3765
Left without being seen					
ED patient volume	Baseline ED LWOBS	Baseline # patient leavers	Improved LWOBS	Improved # of patient leavers	Avoided # of patient leavers
64,000	2.8	1792	2.4	1536	256

Estimates based on an ED annual volume of 64,000 patients. Baseline ED LWOBS estimates are derived from the mean LWOBS at each ED patient volume level. “Baseline number of patient leavers” is derived by multiplying “baseline ED LWOBS” by the annual patient volume. “Improved LWOBS” is derived by subtracting the beta coefficients in Table 2 for ED staffing from the “baseline LWOBS.” “Avoided number of patient leavers” is derived by subtracting the baseline from the improved number of patient leavers.

ED indicates emergency department; LOS, length of stay; LWOBS, left without being seen.

## DISCUSSION

In this large, multistate hospital study, we found that nurse staffing inadequacy in EDs, inpatient units, and at the overall hospital level was associated with worse ED crowding outcomes. ED nurse staffing inadequacy and overall hospital nurse staffing inadequacy were associated with higher left without being seen rates, and ED length of stay for patients with and without behavioral health/psychiatric presentations. Inpatient nurse staffing inadequacy was associated with longer psychiatric/mental health patient length of stay. The findings suggest that improving hospital nurse staffing is a possible interventional target for reducing ED crowding.

Crowding varied significantly across hospitals. For example, left without being seen rates ranged from 1% to 3% (mean 2%), general ED length of stay ranged from 2.5 to almost 4 hours (mean: 3.5 h), and psychiatric/mental health patient ED length of stay ranged from ∼4 hours to 6.5 hours (mean: 5.5 h). These outcomes exceed the targets recommended by the Joint Commission: a rate of <2% for left without being seen, and <4 hours for length of stay (although some organizations recommend <6 h).

The highest percentages of nurses reporting nurse staffing inadequacy were ED nurses (61%); however, over half of nurses in the inpatient (58%) and overall hospital (58%) settings reported staffing inadequacy as well, demonstrating a considerable opportunity for hospitals to improve nurse staffing levels. Nurses’ reports on daily boarding practices also varied across hospitals, with half to all the sampled ED nurses stating that their ED boards patients daily.

Interventions that have been tested to date include rapid medical evaluation areas in EDs, hallway beds in EDs, and inpatient units.^8,10,17^ These interventions are unlikely to be effective in the context of inadequately staffed hospitals with too few nurses to care for additional patients in hallways. In hospitals with sufficient nurse staffing levels, nurses may be able to attend to patients more efficiently in the ED and inpatient units, facilitating patient admissions, discharges, and other essential care measures in addition to more favorable outcomes, including shorter lengths of stay.

We found that nurse staffing inadequacy was high across hospitals, which validates prior evidence demonstrating high nurse workloads before, during, and after the COVID-19 pandemic.^34,35^ ED nurse staffing inadequacy was high in this study, which may contextualize the established evidence that ED nurses experience statistically higher rates of high burnout, job dissatisfaction, and intent to leave as compared with inpatient nurses.^35^ Although nurse job outcomes were beyond the scope of the present study, improving nurse workloads by ensuring that nurses care for a safe patient-to-nurse staffing ratio is an approach that could potentially alleviate ED crowding and, in turn, improve nurse retention in EDs. This inquiry requires further exploration using causal inference approaches.

The results from this study reinforce findings identified in prior literature on nursing resources and ED boarding. Evidence demonstrates that hospitals with safer medical-surgical patient-to-nurse staffing ratios have shorter patient boarding times in the ED.^18^ This study extends the existing evidence by showing that a measure of nurse staffing inadequacy is associated with other measures of crowding, including the rate of leaving without being seen and ED length of stay. Nurses are the clinicians most familiar with patient bedside care experiences, and our findings identify that their reports on boarding are associated with ED crowding. In the context of recent national initiatives to improve reporting of hospital ED crowding measures,^36^ our findings suggest the potential utility of nurses as informants on crowding.

The “Addressing Boarding and Crowding in the ED (ABC-ED) Act”^37^ is a federal bill that was introduced in early 2025 to establish grants for real-time tracking of hospital capacity at the regional and state level across hospitals. This policy does not specify any requirements related to adequately staffing hospitals with nurses, however. It is within hospital jurisdictions to hire enough nurses for safe patient care. In the absence of hospital accountability to staff safety, states have implemented minimum nurse staffing requirements to ensure adequate nurse staffing levels for safe patient outcomes, including California and Oregon.^38,39^ Overall, health policies are needed to reduce ED crowding that encapsulate the real-time monitoring of crowding measures, including adequate nurse staffing levels in hospitals. Our findings contradict fears that minimum nurse staffing requirements would limit patient access to ED care, given that worse staffing was associated with higher rates of patients leaving without being seen and longer ED treatment times.

### Limitations

This analysis used a cross-sectional study design, and thus, causal relationships cannot be assumed. The findings from this study may not be generalizable across all hospitals, given that our study was limited to 10 states and a short period of time. It is well established that hospital crowding fluctuates through seasons of the year,^12^ which we were unable to account for in this study. Thus, the findings may not be generalizable to all hospitals during all periods of time during the year. We did not adjust for patient-level factors that can contribute to ED crowding, given the hospital-level design of the study.

While the measure of nurse staffing inadequacy in this study is nurse-reported rather than an objective FTE, this validated measure allowed for an analysis of nurses’ perceived workload across different nurses’ care settings. Subjectivity bias, influenced by nurse age, years of experience, and recall bias, may have influenced nurses’ reports on this measure. Finally, the aggregation of nurse staffing inadequacy and nurse-reported boarding may contribute to aggregation bias, given that our exposure variable was a hospital-level proportion.

## CONCLUSION

This study identified that inadequate nurse staffing in EDs, inpatient units, and at the overall hospital level is associated with worse ED crowding. Nurses appear to be valid informants of objective hospital crowding measures. Both ED crowding and nurse staffing inadequacy are patient safety threats. Improving nurse staffing is a potential and feasible intervention to reduce ED crowding.

## Supplementary Material
