# Supplementary material for: Nurse Staffing and Emergency Department Crowding: A 10-State Study
Source: Med Care. 2026 Sep 25;64(12):883–9. doi: 10.1097/MLR.0000000000002379 (PMC13641136; doi:10.1097/MLR.0000000000002379)
Supplement: Supplementary file 1 [file mlr-64-883-s001.docx]

**Supplemental File**

**Figure 1: Nurse staffing impact in the input-throughput-output conceptual model**

**INPUT**

Staffing adequacy facilitates:

- timely triage
- notification of providers
- protocol-based care
- rapid medical evaluation

**THROUGHPUT**

Staffing adequacy facilitates:

- Timely receipt of care in ED
- Timely and high-quality care of ED boarders
- Giving report to inpatient units
- Timely receipt of care in inpatient units

**OUTPUT**

Staffing adequacy facilitates:

- Patient education
- Patient discharge
- Timely report to outpatient facilities if needed

**Reprinted from “A conceptual model of emergency department crowding”, Vol 42, Issue 2, Annals of Emergency Medicine, 2003, Asplin et al., with permission from Elsevier.**

**Model Fit Indices:**

**In-text Table 2 Adjusted Models:**

**ED Staffing:**

Left without being seen:

- Adjusted R-Squared: 0.1653
- AIC: 1744.652
- BIC: 1804.894

ED Length of Stay (excluding psychiatric/BH):

- Adjusted R-Squared: 0.2537
- AIC: 4282.122
- BIC: 4342.364

ED Length of Stay (psychiatric/BH):

- Adjusted R-Squared: 0.0825
- AIC: 5153.88
- BIC: 5214.119

**Inpatient Staffing:**

Left without being seen:

- Adjusted R-squared: 0.1375
- AIC: 2452.447
- BIC: 2517.71

ED Length of Stay (excluding psychiatric/BH):

- Adjusted R-squared: 0.2516
- AIC: 5926.21
- BIC: 5991.47

ED Length of Stay (psychiatric/BH):

- Adjusted R-squared: 0.1018
- AIC: 7135.657
- BIC: 7200.92

**Overall Staffing:**

Left without being seen:

- Adjusted R-squared: 0.1289
- AIC: 2582.182
- BIC: 2648.161

ED Length of Stay (excluding psychiatric/BH):

- Adjusted R-squared: 0.2738
- AIC: 6199.462
- BIC: 6265.441

ED Length of Stay (psychiatric/BH):

- Adjusted R-squared: 0.1330
- AIC: 7462.229
- BIC: 7528.21

**Table 1: Sensitivity Analysis using Non-Response Weights**

|  | **Left Without Being Seen (%)** | **ED Length of Stay^1^** | **ED Length of Stay^2^** |
| --- | --- | --- | --- |
|  | **Beta coefficient (95% CI)** | **Beta coefficient (95% CI)** | **Beta coefficient (95% CI)** |
| **ED staffing, unadjusted** | 0.3** (0.1, 0.6) | 11.7*** (6.2, 17.2) | 25.7** (11.2, 40.2) |
| **ED staffing, adjusted** | 0.4** (0.2, 0.6) | 8.5** (3.5, 13.6) | 18.4* (3.6, 33.1) |
| ***Sensitivity analysis*, *adjusted*** | 0.4** (0.2, 0.6) | 8.4** (3.5, 13.6) | 17.5* (3.4, 31.6) |
| **Inpatient staffing, unadjusted** | 0.01 (-0.2, 0.2) | 4.0 (-0.6, 8.6) | 18.8** (6.7, 30.9) |
| **Inpatient staffing, adjusted** | 0.2 (-0.04, 0.4) | 2.5 (-1.8, 6.8) | 18.0** (5.8, 30.3) |
| ***Sensitivity analysis*, *adjusted*** | 0.2 (-0.04, 0.4) | 1.9 (-1.7, 5.6) | 16.5** (4.5, 28.5) |
| **Overall staffing, unadjusted** | 0.1 (-0.1, 0.3) | 7.8** (3.4, 12.3) | 25.0*** (13.5, 36.4) |
| **Overall staffing, adjusted** | 0.3** (0.1, 0.5) | 5.8** (1.7, 9.9) | 22.7*** (11.0, 34.3) |
| ***Sensitivity Analysis, adjusted*** | 0.2** (0.02, 0.3) | 4.7** (1.4, 7.9) | 21.6*** (11.9, 29.9) |

**Notes.** The non-response weights are, for each hospital, the inverse of the probability of responding/reporting each outcome. Such probability is obtained from conducting a logistic regression with outcome a variable indicating whether the “crowding outcome” is available or missing and predictors the hospital characteristics used in our regression models (state, high technology, teaching status, annual ED volume). Such probability is the same as the propensity score, and it is obtained using a non-parametric logistic regression model (i.e.: bosting) as implemented in the R TWANG package.
